# Past vicariance promoting deep genetic divergence in an endemic frog species of the Espinhaço Range in Brazil: The historical biogeography of *Bokermannohyla saxicola* (Hylidae)

**DOI:** 10.1371/journal.pone.0206732

**Published:** 2018-11-05

**Authors:** Augusto César Nascimento, Anderson Vieira Chaves, Felipe Sá Fortes Leite, Paula Cabral Eterovick, Fabrício Rodrigues dos Santos

**Affiliations:** 1 Departamento de Biologia Geral, Universidade Federal de Minas Gerais, Belo Horizonte, Brazil; 2 Programa de Pós-Graduação em Manejo e Conservação de Ecossistemas Naturais e Agrários, Universidade Federal de Viçosa, Instituto de Ciências Biológicas e da Saúde, campus Florestal, Minas Gerais, Brazil; 3 Programa de Pós Graduação em Biologia de Vertebrados, Pontifícia Universidade Católica de Minas Gerais, Belo Horizonte, Brazil; National Cheng Kung University, TAIWAN

## Abstract

The highland endemic frog *Bokermannohyla saxicola* occurs within the Espinhaço Range, the most extensive and continuous orogenic belt of the Brazilian territory, located in southeastern Brazil. We used mitochondrial DNA markers to test for spatial structure, to investigate the likely influence of past vicariant events, to evaluate demographic dynamics along the species range, and to understand the role of habitat discontinuities in promoting connectivity and diversity along the range. We found four major monophyletic lineages, each one associated with distinct mountain tops. The divergence time found between the four main clades clearly pre-dated the Pleistocene, except for the most recent separation. We observed no signs of population expansion for most of the sampling sites along the range, and a higher genetic diversity in the most continuous and central highland plateau, compared to smaller marginal regions. The Espinhaço Range harbors four deeply divergent lineages of *B*. *saxicola* within areas restricted by barriers for millions of years. These relatively isolated populations were kept apart by discontinuities represented by lowland habitats between mountain tops. Most of the lineage divergences occurred earlier than the Pleistocene, thus they cannot be solely explained by climatic oscillations of this epoch. However, within-lineage divergence times were all dated from the Pleistocene, suggesting an important effect in population dynamics. We also suggest that some marginal populations like those from Serra Negra and Serra de Itacambira can be the result of recent colonization events. Finally, in the southern Espinhaço region, the most continuous central highland area shows greater genetic diversity than the marginal discontinuous areas, where we have also observed a significant correlation between genetic and geographic distances. *Bokermannohyla saxicola* is a good model to study the biogeography of the Espinhaço Range because its high genetic structure reflects ancient as well as recent geological/climatic events, with important implications for conservation.

## Introduction

Mountain ecosystems occur on every continent and latitudinal zone of the planet, and are known to harbor remarkable biodiversity [1]. Those located at tropical latitudes are particularly rich in species and usually show high rates of endemism [2–3]. In general, highland habitats have narrow and small areas compared to lowland ecosystems, and are often formed by disjoint units surrounded by habitats with distinctive characteristics [4]. These clusters of discontinuous highlands are often named sky-islands because the intervening environment (lowlands) between them can show remarkable differences in abiotic factors such as temperature, humidity, and soil, as well as species composition [4–7].

Moreover, the population dynamics of highland restricted species can show a high level of complexity as we consider the climatic fluctuations over time. Climate changes are known to have modified patterns of distribution of species and entire biomes across the world, leading to expansion, contraction and latitudinal shifts in the area occupied by them [8–9]. Montane species have also suffered changes in the distribution of their favorable environments along altitudinal gradients [1,8]. In species adapted to specific environmental conditions, populations of different “sky-islands” can experience alternating periods of vicariance and connectivity between them during climatic fluctuation cycles. Periods of connectivity will lead to gene flow between units and expansion towards unoccupied areas, whereas periods of isolation will lead to divergence among populations [9–11]. The effect of these historic events will be reflected in current distribution of genetic diversity and phylogeographic structure of the populations. This complex scenario of diversification, extinction and recolonization, which could lead to distinct outcomes depending on a set of geographical and biological parameters, makes the study of high elevation restricted *taxa* a challenging and attractive field.

The Brazilian territory presents, in general, old terrains that experienced an extensive erosive process for millions of years. Therefore, most of the country is covered by lowland areas, below 1000 meters above sea level (m.a.s.l.). Nevertheless, the territorial relief shows a series of highland areas rising from the prevalent low altitude [12]. Among these, the Espinhaço Range is the largest and most continuous Precambrian orogenic belt of the Brazilian territory, extending in the north-south direction over a length of approximately 1,200 km in eastern Brazil, and covering a great latitudinal range [13]. Its width varies from a few kilometers to a maximum of approximately 100 km, and the elevation reaches up to 2,073 m.a.s.l. These features promote a great diversity of climate conditions and floristic formations.

The montane meadow (*campo rupestre*) is one of the most common habitats occurring in the Espinhaço Range. These habitats can be found, in general, in areas with altitude above 900 m upon quartzite rocky outcrops of Precambrian origin. The typically shallow and oligotrophic soil, together with the seasonally dry conditions, leads to the predominance of open vegetation dominated by herbaceous and shrubby forms [14]. Furthermore, these areas show great heterogeneity of substrates, topography, and microclimate conditions in which different plant families and types predominate [15–17]. The *campo rupestre* was classified as relict vegetation or vegetation refuge by [18], because their floristic composition differs markedly from the floristic context around them. This ecological refuge classification can be extended to the entire habitat since this ecosystem also shows singularity in its fauna, soil and geomorphology [15], when compared to the Atlantic Forest, Cerrado and Caatinga, which are the major ecosystems occurring around the *campo rupestre* of the Espinhaço Range. The *campo rupestre* shelter a great diversity of animal species, and among these, anurans comprise a particularly relevant group with a large number of species (n = 105), and also a high percentage of endemics (27%). Anurans also exhibit great variation in their distributional range along different units of the habitat. Many of them are restricted to specific areas and share this distribution pattern with phylogenetically unrelated species [19], suggesting they were affected by common biogeographic events. The phylogenetic relatedness among the endemic species, and between them and those encountered in the surrounding environments, also raise several questions about the colonization and transition between habitats [4,19]. Thus, the anurans of the Espinhaço Range represent a unique group for biogeographic studies in this habitat. The importance of this group becomes even greater for those interested in phylogeography. Amphibians in general show low individual mobility and phylopatry is often observed in several species. Consequently, populations tend to show higher levels of genetic structure in their spatial range than other vertebrates, and to retain high-resolution signals of the historic events that led the species to the current distribution [20]. Thus, genetic studies of anurans can answer several questions about the history of the *campo rupestre* and reveal not only the relationships between their disjunctive units, but also between them and the surrounding environments along time.

*Bokermannohyla saxicola* is a medium size (50–54 mm snout-vent length) anuran that inhabits and reproduces in permanent streams with rocky bottoms, where larval development takes at least five months [21]. The species is endemic to the *campo rupestre* of the Espinhaço Range in Minas Gerais state, Southeastern Brazil. Its distribution ranges from Barão de Cocais municipality, in its southern limit, to the northern edge of Minas Gerais state, where a great discontinuity separates it from the northernmost portion of the Espinhaço Range (known as Chapada Diamantina) located in Bahia state [19]. *B*. *saxicola* usually occurs above 800 m.a.s.l., associated to small rocky streams, which are rarely found below this elevation. However, in rare instances, such small rocky streams occur in lower elevations, usually on the Atlantic slopes of the Espinhaço Range. Thus, the species has already been recorded around 600 m.a.s.l. This lowest elevation limit does not to seem to vary latitudinally across the species distribution (Leite, F.S.F., personal communication).

A preliminary study identified population differentiation based on microsatellites and high allelic diversity in *B*. *saxicola* [21]. Given the small spatial scale in what this study was conducted, the levels of genetic structure can be considered above the expectations. A larger scale study is then needed to better understand and characterize the relationships between the current demes and the history of the species. This becomes especially relevant due to the lack of studies concerning the genetic status of animal species endemic to the Espinhaço’s *campo rupestre*.

Here we aim to examine the phylogeographic structure present in *Bokermannohyla saxicola* using mitochondrial DNA sequences from individuals sampled across its entire distribution. By considering characteristics of this distribution such as continuity and area size of land masses, as well as the likely effects of past climate changes in the configuration of the environment in eastern Brazil, some hypotheses were raised and tested about the historic and current distributions of the species diversity. First of all, we considered ecological questions raised by the configuration of the elevated areas of the Espinhaço Range. In its southern portion, the range presents an almost completely continuous, large and wide zone above 1000 m.a.s.l., ranging from the Serra do Cipó to the Diamantina Plateau region in Minas Gerais state. The other areas in which the species is found are, in general, narrower, shorter, and more discontinuous. They are also separated from the southern continuous region by lowland areas of distinctive habitat. Here we test the hypothesis that the lowland regions work as barriers to the dispersion of the individuals. Another hypothesis can follow from the previous. The populations at the southern core region in Espinhaço Range present a larger area of suitable habitat. Furthermore, if the lowlands are barriers, the populations located in the larger area are expected to show higher rates of gene flow among subpopulations. Together, these factors can result in higher levels of genetic diversity in the continuous region when compared to the isolated, discontinuous or marginal zones at the edges of the distribution [22,23]. We also considered the effects of the Pleistocene climate changes in the connectivity of populations between actually separated areas, testing the hypothesis that differentiation between *B*. *saxicola* populations located at distinct habitat units occurred during the warmer periods between glaciation times.

## Materials and methods

### Ethics statement

This genetic study was based on biological material previously collected and deposited in the Centro de Coleções Taxonômicas da Universidade Federal de Minas Gerais (CCT-UFMG).

We use only the CCT collection tissue and there were no specific collections for this project. Institutions of the Brazilian Government that granted research, fieldwork and collection permissions for these samples were Instituto Chico Mendes de Conservação da Biodiversidade (ICMBio) and Instituto Estadual de Florestas (IEF). Specimens were collected for other studies by B. G. Pacheco and P. C. Eterovick (permanent collection permit for PCE: Sisbio/ICMBio10748-1), F. F. R. Oliveira and P. C. Eterovick (permits IEF 085/06 and Sisbio/ICMBio 12813–1) and F. S. F. Leite (permits Sisbio/ICMBio 21185–1). Many individuals were tadpoles and were used for fluctuating asymmetry measurements [24] that turned them unsuitable for museum collections. Other are still being used for other studies by B. G. Pacheco and PCE. These collection permits supported the following collection method: tadpoles were euthanized with topical pure benzocaine 10% and preserved in formalin 10% immediately after collection. No additional information on Animal Care and Use Committee is linked to the institutional repository of these samples in the CCT database.

### Sampling and sequencing

**Fig 1:**
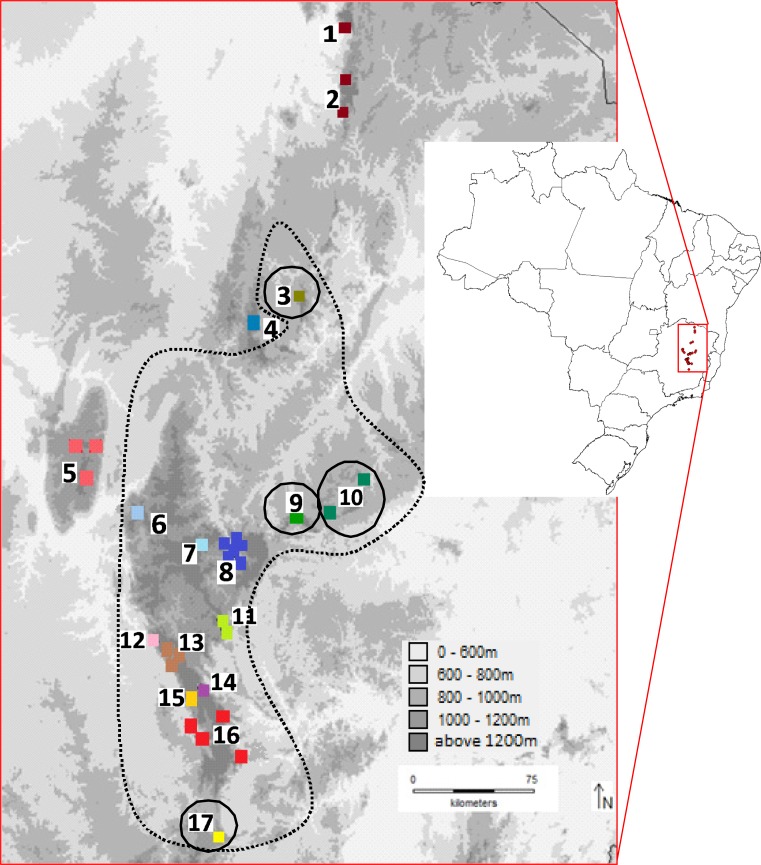
Sample sites across the Espinhaço Range. The Southern Espinhaço region is within the dotted line. The sample points in the marginal areas are circulated. 1: Serra da Formosa; 2: Serra Nova; 3: Serra de Botumirim; 4: Serra de Itacambira; 5: Serra do Cabral; 6: Águas de Santa Bárbara; 7: Diamantina; 8: Rio Preto; 9: Serra do Ambrósio; 10: Serra Negra; 11: Serra do Sapo; 12: Alto de Fechados; 13: Serra Talhada; 14: Conceição do Mato Dentro; 15: Lapinha da Serra; 16: Serra do Cipó; 17: Serra da Água Limpa.

We obtained *Bokermannohyla saxicola* samples from 34 localities in 17 main mountains across its distribution throughout the Espinhaço Range (Fig 1 and S1 Table). The supplementary material S2 Table contains specimen numbers and Genbank accession numbers of the all samples used in this study and deposited in the CCT-UFMG. The sampling points obtained are representative of the entire latitudinal range and most of the total known distribution of the species. A total of 220 individuals were obtained with a range of 1–20 individuals per site (see S2 Table). Tissues were sampled from tadpole tails, adult liver or muscle samples and preserved in 96% ethanol before DNA extraction. For DNA extraction we used a phenol–chloroform–isoamilic alcohol protocol [25].

We amplified and sequenced a fragment of two mitochondrial coding genes, *Cytochrome b* (*Cyt-b*) and *Cytochrome c Oxidase subunit I* (*COI*), using published primers CYTB2F and CB3-H [26] for *Cyt-b*, and LCO1490, HCo2198 [27], Chmf4 and Chmr4 [28] for *COI*. The amplified fragments consisted of approximately 700 bp. The polymerase chain reactions (PCRs) were done under the following conditions: 94°C for 5 min, 35 cycles of 51°C (LCO-HCO), 53°C (CB3H-Cytb2f) or 56°C (Chmf4-Chmr4) for 40 s, 72°C for 1 min, 94°C for 40 s and a final extension of 10 min at 72°C. The detailed protocols for PCR amplification and sequencing were performed as in [29].

Contig alignments were obtained using the programs phred version 0.20425 [30], phrap version 0.990319 (http://www.phrap.org) and consed version 14.0 [31]. The alignments of the consensus sequences for all individuals were built using clustal w algorithm available in the software MEGA 6 [32].

### Phylogenetic, molecular dating, and phylogeographic analyses

We concatenated the sequences of the two mtDNA genes to generate haplotypes for each individual and used DNAsp v5.10.1 [33] to identify unique haplotypes in the dataset. Molecular dating and phylogenetic relationships among the haplotypes were obtained by Bayesian inference performed in BEAST v1.8.2 [34]. Prior substitution rates were set as lognormal distributions according to estimations for a clade of European-Asian frogs of the genus *Hyla*, that belongs to the same subfamily as *Bokermannohyla*. For the *Cyt-b* gene we used the rate estimated by [35] (mean = 0.0161, SD = 0.07). For the *COI* gene we used the rate (mean = 0.020321, SD = 0.15) we estimated for the same *Hyla* clade using the divergence times in [35] and *COI* sequences from [36]. The best fit evolutionary model for each gene was determined using jModelTest [37] and selected based on the Bayesian information criterion (BIC). We employed the TN + G evolutionary model for the *COI* fragment and HKY+G+I for *Cyt-b* as indicated by Modeltest and used a Birth-death process as tree prior. We performed two independent analyses that ran for 1.0 x 10^8^ generations, sampled every 1000th generation. Stationarity was checked visually using a 10% burn-in in Tracer 1.5.

The *COI* rate estimation was performed in BEAST 1.7 [34] with the HKY model for sequence evolution and an uncorrelated relaxed lognormal clock. The age priors were set as lognormal distributions with means and standard deviation values that matched the estimates for *Cyt-b* in [35] accordingly. The analysis ran for 3.0x10^7^ generations, sampled every 1000th generation, and we used a 10% burn-in after checking for stationarity in Tracer 1.5. The concatenated haplotypes were later used to construct a median-joining network in the software Network version 4.611 [38] for each of the major clades detected in the Bayesian tree.

### Diversity indices and population differentiation

The sampling sites were first divided according to the four main clades observed in the phylogenetic trees and analyzed separately because of the high divergence among them. Localities with fewer than six individuals were removed from these analyses. Sampling sites within less than 2 km of distance were grouped together. In the northernmost clade, due to the lack of amplification of the *Cyt-b* fragment in several individuals, we used only a 546 bp *COI* fragment. This clade was divided in three sampling sites for analyses. Itacambira clade was considered one single population because it consisted of only one sampling site. The “Serra do Cabral” clade was excluded from these analyses because there were too few individuals.

Estimation of diversity indices, neutrality tests and population structure analysis were performed in Arlequin v3.5 [39]. To determine genetic diversity across the species range we estimated allelic (*s*) and nucleotide diversity (*π*) at each sampling site. We accessed demographic history using Tajima’s *D* and Fu’s *Fs*. Population pairwise genetic differentiation was quantified by *ΦST* between sampling sites using 10,000 random permutations.

The tests for genetic diversity differences and isolation by distance (see below) were performed only on the populations belonging to the southern clade. This choice aimed to reduce the influence of historical events, once the divergence times between clades were high. This clade was chosen because it comprises most of the species range and sampled localities. To evaluate the isolation by distance and the effects of discontinuities on population differentiation we compared the results of Mantel tests using two datasets with different combinations of sampling sites. All tests were performed in the IBD software [40] using 1000 permutations. The first combination included all sample sites (with 7 or more individuals) within the southern Espinhaço region, and the second included only sites within the continuous core region. The ΦST values were linearized using [41]’s equation. We also analyzed two datasets of geographic distances. Initially, we measured the absolute distances between each pair of sampling points. In the second dataset, we connected each point to its nearest neighbor, except for Serra de Botumirim, that was connected to one of the sites at Rio Preto because it was the nearest point in the continuous core region. The connections presented a shape near to a 1D stepping-stone model, with only one bifurcation at the Rio Preto 2 site. Distances between sites were measured on this shape. This correction was made because straight lines connecting some sites presented unrealistic pathways crossing long distances of unsuitable habitats.

To investigate the location of possible barriers to gene flow, we used Monmonier’s maximum difference algorithm as implemented in the software Barrier 2.2 [42]. To compare the genetic diversity of central and marginal populations we estimated haplotype and nucleotide diversity for each site. We also estimated effective population sizes for the sample sites using Migrate [43]. All migration rates were set to zero in the parameter matrix. The analysis was conducted in a Bayesian framework using ten short MCMC chains with 1000 iterations and one long chain with 1x 10^6^ iterations and 10% burn in. As we do not know the generation time of the species to convert the mutation rate from years to generations, the parameter *θ* (2*Neμ*) was used for later analyses. We performed a one tailed Wilcoxon rank sum test for each variable in R [44]. The alternative hypothesis was that diversity, or effective population size (*θ*), are greater in the center than at the margins of the species distribution.

## Results

### Phylogenetic, phylogeographic and molecular data analyses

**Fig 2:**
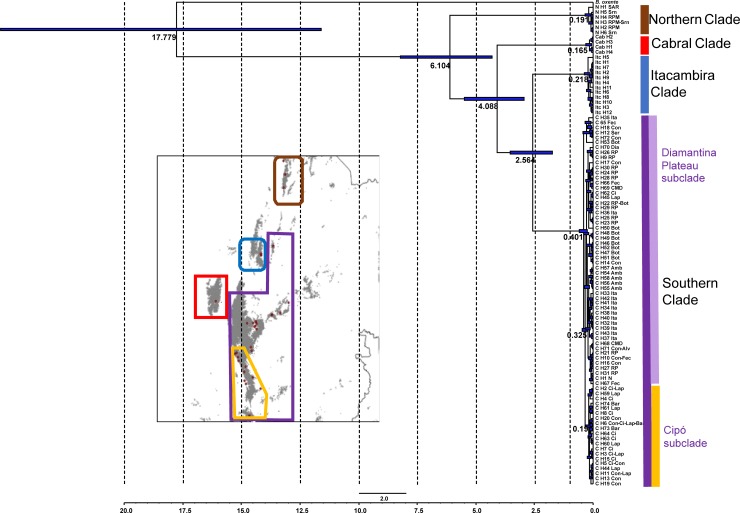
Divergence times and phylogenetic relationships within *B*. *saxicola*. Bayesian Tree with divergence times and phylogenetic relationships within *B*. *saxicola* complex haplotypes. The blue bars represent the divergence time with the 95% HPD confidence interval. The map indicates the four main clades, and the two subclades.

The phylogenetic tree (Fig 2) showed high resolution in the older splits (posterior probability >0.9), although it presented mostly low values of posterior probability (<0.9) in the newer ones. The tree consisted of four main clades with high posterior probabilities and millions of years of divergence among them. These clades showed a clear geographical correlation. The most basal clade (Northern) contains the haplotypes restricted to the three northernmost localities. The next clade to diverge from the others presents haplotypes found only at Serra do Cabral. The next lineage divergence separates a clade encountered only at Serra de Itacambira from the major clade (Southern), that covers most of the species range, in the southern part of its distribution.

**Fig 3:**
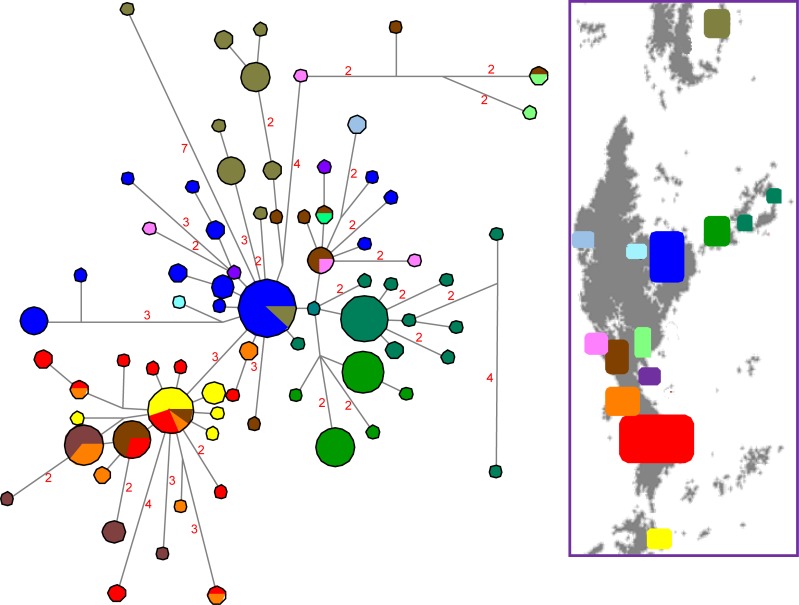
Median joining haplotype network of *B*. *saxicola* Southern clade. Legends for the colors of locations are indicated in the map. The number of substitutions is proportional to the length of the line connecting haplotypes and is also shown, except when there is only one mutational step. The size of the circle is proportional to the frequency of the haplotype obtained in the sample.

The Southern clade contains two relatively large subclades with high posterior probabilities. The southernmost (Cipó subclade) is restricted to an area ranging from Serra Talhada, at Congonhas do Norte municipality, to the southernmost border of the distribution. The other subclade (Diamantina Plateau subclade) is mainly found in the northern part of this area, except for a few haplotypes found at Serra do Cipó, and Serra Talhada, where both subclades occur in similar frequencies. The two subclades of the Southern clade are separated by only three mutation events in the haplotype network (Fig 3). In this clade, only eight of the 74 haplotypes are shared between different localities.

Other two clades also present internal subdivisions. In the Northern clade, the single haplotype found in Serra da Formosa is separated by four mutational events from the ones found at the other two localities. Four mutation steps also separate the Cabral clade in two groups that are not found at the same sampling site.

According to our time estimates, the origin of the *B*. *saxicola* taxon is dated to about c. 6.1 million years ago (Mya), during the Late Miocene, when the Northern clade diverges from the others. The sequential separation of the clades in the species tree indicates a north to south dispersion, where the Northern clade is the first to separate, followed by Cabral, Itacambira, and Southern clades. The Early Pliocene marks the separation of the Serra do Cabral lineages from other clades at c. 4.1 Mya. The last major divergence occurred between Itacambira and the Southern lineages, which occurred at c. 2.56 Mya in the Early Pleistocene. The intra-clade diversification of the four major lineages of *B*. *saxicola* occurred during the Pleistocene, *circa* 325 Kya for separation of subclades Cipó and Diamantina Plateau in the Southern clade, and *circa* 218, 165 and 191 Kya for Itacambira, Cabral and Northern clades, respectively. The average divergence time estimates among the main clades were mostly older than the Pleistocene, except for the most recent divergence observed between Southern x Itacambira clades. Furthermore, intervals for the time to the most recent common ancestor of all lineages within the main clades were all younger than 500 thousand years ago (Kya).

### Diversity indices and population differentiation

Diversity indices showed great variation across the range of the species (Table 1). The continuous region in the south of the distribution, together with the Serra de Itacambira and Serra de Botumirim sampling sites, showed the highest values for haplotype and nucleotide diversity. Diversity in all the other regions presented, in general, smaller values. Serra da Formosa showed the lowest diversity, as all the individuals had the same haplotype. In the Southern clade, populations located in the continuous core region showed significantly more haplotypes and nucleotide diversity than in the discontinuous or marginal regions. The Wilcoxon test for higher diversity in the continuous region showed significant results for both haplotype and nucleotide diversity indices (p = 0.017 and p = 0.015 respectively). Even though the continuous region of the southern Espinhaço presented, in general, higher *θ* values, global differentiation among sampling sites was not significant (p = 0.06).

**Table 1:** Sample size, estimates of haplotype (*H*) and nucleotide diversity (*π*), Tajima’s *D* and Fu’s *Fs* results for the sample sites with six or more individuals.

Sampling site	Sample size	*H*		*π*	Tajima's *D*	*FS*	*θ*
Serra da Formosa	20	0		0	0	0	0.0015
Serra Nova1	6	0.6		0.002198	1.75324	1.93787	0.00089
Serra Nova2	10	0.8		0.004518	-0.01324	-0.12561	0.00148
Serra de Itacambira	20	0.9263		0.003804	-1.03898	-3.83766*	0.0005
Serra de Botumirim	19	0.8889		0.003762	-1.02385	-1.13845	0.007
Serra Negra1	7	0.5238		0.001713	-1.52412*	1.01423	0.00396
Serra Negra2	16	0.8667		0.002564	-1.69314*	-4.48941*	0.00894
Serra do Ambrósio	20	0.6632		0.001935	-0.0604	0.60012	0.00289
Rio Preto1	9	0.8889		0.002498	-1.49216	-1.6034	0.00814
Rio Preto2	9	0.8333		0.002941	-1.28067	-1.20619	0.00124
Serra Talhada1	10	0.8889		0.003907	0.02622	-0.18799	0.00949
Serra Talhada2	10	0.9333		0.006682	-0.84021	-1.23689	0.01101
Lapinha da Serra	13	0.9359		0.003894	-1.02552	-2.57562	0.01067
Serra do Cipó	7	0.9524		0.003806	-0.81948	-1.69576	0.01106
Serra de Água Limpa	10	0.6		0.000666	-0.18393	-0.27178	0.00129

* Significant values are indicated by an asterisk.

Pairwise *ΦST* showed highly significant values in most of the comparisons (Table 2). All the comparisons between sampling sites located less than 7 km from each other resulted in non-significant and low *ΦST* values. The two sampling sites at Serra Negra, municipality of Itamarandiba, also showed a non-significant value, although they are separated by approximately 20 km of lowland areas. The Lapinha da Serra region, located between Serra do Cipó and Serra Talhada, showed low and non-significant values when compared to these two neighboring regions, although Serra do Cipó and Serra Talhada yielded a significant *ΦST* value between them. In the Northern clade, Serra Nova sites showed no significant differentiation between them, but each site presented significant differentiation values when compared to Serra da Formosa.

**Table 2:** Differentiation between *B*. *saxicola* populations and their spatial distribution. Upper diagonal shows absolute distance values in Km. Lower diagonal shows pairwise *ΦST* values between sample sites with more than six individuals in the Southern clade.

		Am	Ba	Bo	Se	Co1	Co2	It1	It2	La	Ri1	Ri2
**Serra do Ambrósio**	**Am**	0	203.7	138.6	148.3	113.9	118.7	46.3	20.1	133.6	38.5	38.9
**Serra de Água Limpa**	**Ba**	0.7845	0	340.4	63.7	119.2	111.7	239.6	213.6	82.2	182.7	187
**Serra de Botumirim**	**Bo**	0.5527	0.6035	0	281.5	236	242.7	118.4	174.8	264.7	159.1	155.4
**Serra do Cipó**	**Se**	0.7106	0.1637	0.5387	0	55.8	48.3	178.9	151.5	18.7	122.4	126.3
**Serra Talhada1**	**Co1**	0.6103	0.237	0.4312	0.1548	0	7.6	159.3	131.2	37.5	79.7	82.4
**Serra Talhada2**	**Co2**	0.5103	0.1827	0.3417	0.1242	-0.06*	0	163.7	135.5	30.2	85.5	88.4
**Serra Negra1**	**It1**	0.5724	0.8242	0.4733	0.6548	0.5261	0.3769	0	28.2	171.8	84.5	84.3
**Serra Negra2**	**It2**	0.4986	0.708	0.4477	0.615	0.491	0.3795	-0.023*	0	143.8	58.3	58.9
**Lapinha da Serra**	**La**	0.6459	0.1097	0.4798	0.0615*	0.0009*	0.0254*	0.5747	0.5472	0	105.9	109.3
**Rio Preto1**	**Ri1**	0.5452	0.6739	0.2645	0.5221	0.3715	0.225	0.489	0.3784	0.42	0	4.9
**Rio Preto2**	**Ri2**	0.4963	0.6453	0.259	0.5016	0.3354	0.201	0.401	0.3001	0.4082	-0.021	0

* Non-significant values are indicated by an asterisk.

We found correlation between genetic and geographic distances in both datasets analyzed. The dataset containing only the sampling sites within the continuous region presented a much higher correlation index than the dataset including the complete set of sampling sites of the southern Espinhaço in every combination (Fig 3). The tests using the corrected distances yielded slightly higher correlation indexes than the ones using absolute distances. The discrepancy in these results was higher in the analyses using the complete list of sampling sites.

**Fig 4:**
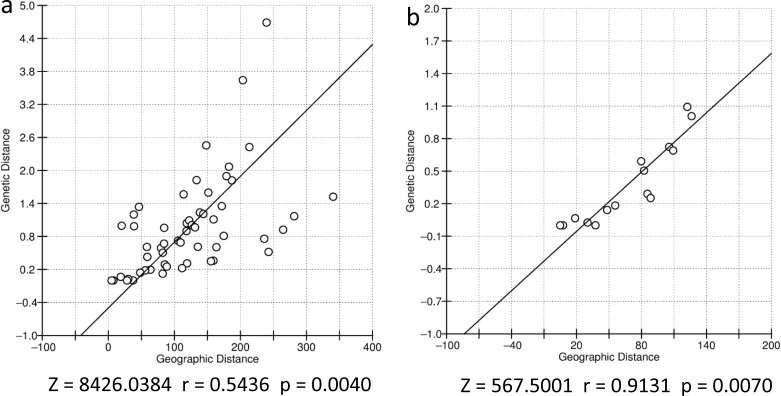
Mantel test results. Correlation coefficient values and p-values for the null hypothesis of r ≤ 0 are indicated below the graphics. A) Complete southern clade. B) Continuous core region in southern Espinhaço.

Several discontinuities across the species range were detected with Barrier software. The first five barriers to gene flow identified in this analysis were correlated with geographical features along the southern Espinhaço (Fig 4). Most of them coincided with areas below 1000 m.a.s.l. and separated the regions defined as marginal areas from each other and from the continuous region.

Demographic analysis performed with neutrality tests yielded diverse results for sampling sites. In the southern Espinhaço, only the sampling sites at Serra Negra had a significant result for at least one test (p<0.05). The Itacambira clade had significant results only for Fu’s *Fs*. In the northern region, no sample site revealed significant signs of expansion (Table 1).

## Discussion

### Biogeographic history of *B*. *saxicola*

The ability and accuracy to provide molecular dates for diversification events permits to draw correlations with past geological and climatic events, and thus are essential to make good inferences biogeographical. Thus, as we use substitution rates estimated for European tree frog lineages, this may result in a partial inaccuracy in our chronology ages for *B*. *saxicola*. Differences in parameters as DNA repair efficiency, population size, metabolic rate and generation time are among the most likely sources of among-lineage rate heterogeneity [45], mainly due to the evolutionary and population histories intrinsic to each species. However, substitution rates are likely to be similar in closely related species, such as clades should experience similar rates of molecular evolution [45]. In our study, we sought to use the most parsimonious substitution rates for the Hylidae family, due to the lack of frogs’ models for molecular clock calibration from Neotropical region. In addition, the recent published phylogeography for another endemic frog from the Range Mountain Ridge, *Pithecopus megacephalus* [46] did not new estimate substitution rates and acts priors for mitochondrial genes. Thus, our analyzes were conducted in the best possible way in the current scenario.

The estimated times for the origin and dispersal of *B*. *saxicola* lineages can be compared to the available paleoclimatic data for the Espinhaço Range. Palinological analyses of sediment cores dating from the last glaciation, sampled from elevated areas in southeastern Brazil, show the dominance of grassland taxa until the end of the last glaciation in the Pleistocene/Holocene boundary [47–49]. Along with other data from all over South America, this suggests a cooler and drier climate during the glaciations, becoming warmer and wetter towards the beginning of the Holocene. So, there is a strong evidence for the expansion of elevated grassland habitats during the last glaciation, when currently isolated areas were probably linked by similar environments [49]. The Pleistocene epoch was marked by several glaciation cycles, with repeated alternating climate scenarios that probably provided many opportunities for migration and colonization, as well as vicariance events. However, there are some aspects we should take into account for this particular highland endemic species. First, considering only temperature, we should expect a larger distribution range for this frog during the colder climate of the glaciations, and a more fragmented distribution in the interglacial periods, like now in the Holocene. But this species, which reproduces in permanent streams, may also be severely affected by humidity and water supply, and Pleistocene glaciation episodes were usually associated with low precipitation worldwide. Thus, even though glacial periods of the Pleistocene may favor connection between highland populations because of overall low temperature, this period could be also much drier, perhaps limiting and decreasing populations in some regions across the Espinhaço Range.

Many species endemic to the Espinhaço Range, as *Eurolophosaurus nanuzae*, *Hypsiboas cipoensis* and *B*. *saxicola* could have originated by isolation during maximum moisture periods in the Quaternary, likely during interglacial episodes, when these open areas probably were isolated by forests [50,51]. However, considering only the deep divergence found between the four main clades of *B*. *saxicola*, the Pleistocene climatic oscillations do not seem to be directly involved, as the confidence intervals for the two oldest divergence times fall clearly before the beginning of the Pleistocene. On the other hand, the median of the divergence time for the youngest clades (Southern and Itacambira) falls in the early Pleistocene. Although the lower limit of its confidence interval (CI) extended almost one million years within the Pleistocene, its upper limit extends nearly the same amount of time before in the Pliocene. Much importance is given only to the recent events of the Quaternary on the diversification of South American herpetofauna, rather than Tertiary events [50,52] which, in turn, probably could have a much greater importance than assumed in diversifying herpetofauna of South America.

The earlier divergence estimated between the Northern clade and the rest of the lineages present a confidence interval encompassing the Late Miocene and Early Pliocene. Some climatic oscillations in these epochs could be raised as explanations for this ancient divergence. For example, the Late Miocene ranging from 6.3 to 5.3 Mya was marked by 18 glacial-to-interglacial oscillations [53,54], when population range shifts due to climate changes could be the cause of past vicariance. This basal split is also correlated with the greater spatial discontinuity in the distribution of the highland areas.

Although Serra do Cabral is spatially closer to the most continuous southern highland island than to Serra de Itacambira, Itacambira and the Southern clade share a closer phylogenetic relationship. This could be explained by characteristics of the terrain between them. The separation between Itacambira and the southern plateau presents areas below 1000 m.a.s.l., but they are not as low as the ones separating Serra do Cabral from the southern continuous region, which vary from 550–600 m.a.s.l. Another evidence that indicates this lowland region between Itacambira and southern area to be a less effective barrier is the population of Serra de Botumirim. Even though Botumirim bears haplotypes belonging to the Southern clade, this area is located even further north than Serra de Itacambira. This shows that the species was capable of crossing these particular lowland regions in recent times.

The confidence intervals of the two most recent divergence times cover, at least in part, the Pliocene epoch. This epoch is known for being usually warmer than the Pleistocene. A particularly warm and stable period happened from 3.6 to 2.6 Mya, in Late Pliocene, when Earth temperatures were, in average, 2–3°C higher than today [55,56]. Many shifts towards higher latitudes in the subtropical and tropical forests are documented for this warm period [57], thus warm climate in this epoch could have led to retraction of *B*. *saxicola* populations to the higher peaks of the Espinhaço Range, leading to vicariance events. The divergence time between Serra do Cabral and others (Southern + Itacambira) can be notably associated with the Late Pliocene vicariance. On the other hand, the divergence time between Serra de Itacambira and Southern clades may be associated with the interglacial periods in the Early Pleistocene [56,58]. These glaciations occurred in 40,000 year cycles and are known for being shorter than the 100,000 year cycles of the Late Pleistocene [58–60].

Population divergences within all clades occurred in the Late Pleistocene, because all confidence intervals for the most recent common ancestor dated to less than 500 Kya. This period is known for a shift in the temperature of interglacials, being warmer than those of the Early to Middle Pleistocene [61].

### Population structure and genetic diversity

The clades of *Bokermannohyla saxicola* show strikingly ancient divergence times for such a small distribution range, although similar or even older ages can be found within anuran species [62–66]. The sky-islands of the Espinhaço Range seem to have worked as sanctuaries, keeping alive descendants of ancient divergent lineages despite climatic variations over time. Some of these clades are restricted to areas less than 100 km long. The presence of extensive lowland areas among geographically delimited mountain top areas where the four main clades occur shows clearly that lowlands are effective barriers to dispersion, preventing gene flow (at least maternal) for millions of years. This is not an uncommon scenario for species restricted to mountain regions. The role of the lowlands between highland massifs in the segregation of distinct lineages was evidenced even in vertebrate groups with a higher dispersion ability [67–68]. In mountain-endemic amphibians, divergence times between lineages as high as 6 and 8.5 Mya were already found [66,69].

In the area occupied by the Southern clade, the genetic diversity of a sampling site or population is influenced by its position in the distribution. Populations situated in the discontinuous or marginal regions show significantly less haplotypes and nucleotide diversity than the ones located in the continuous core region. This continuous region also exhibits a noteworthy correlation between genetic and geographic distance, much more intense than the clade as a whole.

The areas in the south portion of the Southern clade distribution (Serra da Água Limpa, at Barão de Cocais municipality, Serra do Cipó and Lapinha da Serra) were probably colonized originally by members of the Cipó subclade, as only three individuals in the whole region present a haplotype belonging to the Diamantina Plateau subclade. Except for Serra Talhada, all the other sampled regions in the north part of the Southern clade range contain only haplotypes from the Rio Preto subclade. The Diamantina Plateau subclade is clearly separated from the Cipó subclade in the haplotype network and shows clear association with the north area of the Southern clade distribution. Thus, we identified distinct founders for northern and southern areas where the Southern clade occurs. The haplotype network shows many haplotypes derived from the one found almost only in the Parque do Rio Preto region. This is an evidence that this region could be probably the source of this subclade. Serra Talhada is the only region that presents haplotypes of both subclades in similar frequencies, working as a secondary contact area, likely resulted from the dispersion of Diamantina Plateau and Cipó subclades coming from north and south, respectively.

Geographic position along a species range is expected to influence genetic diversity of the populations. This is the scenario found for the southern Espinhaço, where core areas apparently show greater habitat suitability and connectivity, leading to higher diversity. The decline of appropriate habitat conditions towards the margins leads to decreased gene flow between populations and smaller effective population size, implicating a reduction of genetic diversity [22,70,71]. As most of the main discontinuities detected using the Barrier software separated the marginal populations from the continuous region, the hypothesis of lower gene flow in marginal areas is strongly supported. We found correlation between geographic and genetic distances in the southern Espinhaço, notably in the continuous highland region. Nevertheless, current isolation by distance (IBD) does not appear to be the main cause of this scenario. IBD occurs due to a contemporary balance between local genetic drift and geographically mediated gene flow [72]. The presence of distinct phylogenetic mtDNA clades, many geographically exclusive haplotypes and high population pairwise ΦST suggest a more complex scenario. The main process behind it is probably the result of a relatively long term structured divergence in space under influence of current and historical events detected in this study.

Significant neutrality test results for the Itacambira and Itamarandiba regions suggest that they were originated by recent colonization events, followed by demographic expansions. Climatic changes since the beginning of the Holocene could have changed the distribution of suitable habitats at these areas favoring their colonization and leading to range shifts towards the current distributions.

The southern Espinhaço presented the greatest diversity of haplotypes, even though this could be also the result of a larger area of occurrence and the extension of sampling. The few substitutions separating haplotypes from each other suggest that expansion and colonization in these areas could be a recent event. However, the high frequency of exclusive haplotypes indicates that the populations had substantial divergence time to accumulate these singular haplotypes. The usually high population pairwise ΦST values detected across the distribution strengthens the hypothesis of an earlier divergence through relative isolation and genetic drift. This divergence becomes especially clear in the northern populations of this area, where some sampling sites present only exclusive haplotypes, as Serra do Ambrósio and Serra Negra. The Serra de Botumirim and Rio Preto regions share a single haplotype, and all the other haplotypes are restricted to one of them. This haplotype distribution is probably the effect of the geographic configuration of the area. In this zone, the sampled regions are discontinuous, clearly separated by lowland habitats, where barriers to gene flow were detected (see S1 Fig). Thus, the presence of lowlands also seems to be the most likely cause of population divergence in a recent time and in a microgeographic scale, as it happened during the divergence of the main clades for millions of years.

The presence of two distinct subclades of haplotypes in the Southern clade indicates a north-south vicariance event in the Middle Pleistocene. The average date for the divergence between the subclades falls near the MIS 9 interglacial (320 Kya), a period of warmer temperatures than the Holocene [73]. The areas below 1200 m.a.s.l. observed between Serra Talhada and Rio Preto, which was also highlighted by the Barrier software (S1 Fig), could likely be the barrier causing this segregation.

Climate changes raise questions about the stability of the populations. The marginal areas of a species distribution tend to have less suitable habitats, and to suffer frequent extinctions and recolonizations. The recolonization is often made by a few founding individuals, and is followed, in general, by a population expansion event when they reach the suitable habitat. On the other hand, core populations tend to have a stable size over time, determined by their carrying capacity. Given the likely historic scenario of the montane meadows of southeastern Brazil, range shifts driven by climatic changes could have led to recent colonization in some areas, which present signs of demographic expansions.

### Systematic, taxonomic and conservation implications

The deep divergence observed among clades of *B*. *saxicola* has taxonomic and conservation implications. Although morphological differences have not been previously reported in the whole range of the species, our data shows it is clearly divided into four distinct clades highly structured across its spatial distribution. These results draw attention to the importance of comparative investigations using also morphological and behavioral characters, like the structure of mating calls, along the species range. Such studies are necessary to determine whether the mtDNA clades can represent distinct species. Even if the populations of different mtDNA clades are not shown in the future to be different species, they can be at least considered as four different evolutionary significant units for conservation purposes. If one of the main clades restricted to a small and threatened area like the Serra do Cabral confirms to be a new species, it could be easily assigned as an endangered taxon. The type location for *B*. *saxicola* is Serra do Cipó, part of the area where the main Southern clade occurs, which would retain the original species name [74]. Even though this clade exhibits the largest range of all, its distribution would be far less than the currently assigned species distribution, thus the conservation status of *B*. *saxicola* should be reevaluated.

Our results indicated a remarkable population structure across the spatial range of *B*. *saxicola*, which seems to have been caused by past vicariant events affecting habitat availability/connectivity. Besides, we show this endemic species is a very interesting model for testing alternative vicariance scenarios in the Espinhaço Range, dating back to ancient divergence during the Pliocene.

## Supporting information

S1 Table Sampling localities in the Espinhaço Range. (DOC)

S2 Table Genbank accession numbers of *B. saxicola* samples used in this study. (DOC)

S1 Fig Barriers identified for *B. saxicola*. Voronoi tessellation and Dealuney triangulation superimposed to the sample map. The five barriers are identified by alphabetical order at the edges of each barrier.
(TIF)
