# Supplementary material for: Past vicariance promoting deep genetic divergence in an endemic frog species of the Espinhaço Range in Brazil: The historical biogeography of Bokermannohyla saxicola (Hylidae)
Source: PLoS One. 2018 Nov 5;13(11):e0206732. doi: 10.1371/journal.pone.0206732 (PMC6218059; doi:10.1371/journal.pone.0206732)
Supplement: S1 Table — (DOC) [file pone.0206732.s001.doc]

**S1 Table. Sampling localities in the Espinhaço Range.**

| **Code** | **Municipality** | **Locality** | **Latitude** | **Longitude** |
| --- | --- | --- | --- | --- |
| *Bokermannohyla saxicola* | |  |  |  |
| 1 | Santo Antônio do Retiro | Serra da Formosa | -15.250 | -42.814 |
| 2 | Rio Pardo de Minas | Serra Nova 1 | -15.610 | -42.810 |
| 3 | Serranópolis de Minas | Serra Nova 2 | -15.790 | -42.810 |
| 4 | Botumirim | Serra de Botumirim | -16.843 | -43.063 |
| 5 | Itacambira | Serra de Itacambira | -16.991 | -43.317 |
| 6 | Joaquim Felício | Serra do Cabral | -17.672 | -44.225 |
| 7 | Francisco Dumont | Serra do Cabral | -17.685 | -44.323 |
| 8 | Itamarandiba | Serra Negra 1 | -17.806 | -42.656 |
| 9 | Buenópolis | Serra do Cabral | -17.860 | -44.250 |
| 10 | Itamarandiba | Serra Negra 2 | -18.005 | -42.748 |
| 11 | Augusto de Lima | Águas de Santa Bárbara | -18.031 | -44.063 |
| 12 | Rio Vermelho | Serra do Ambrósio | -18.089 | -43.072 |
| 13 | São Gonçalo do Rio Preto | Rio Preto | -18.125 | -43.357 |
| 14 | São Gonçalo do Rio Preto | Rio Preto | -18.126 | -43.357 |
| 15 | São Gonçalo do Rio Preto | Rio Preto | -18.129 | -43.370 |
| 16 | São Gonçalo do Rio Preto | Rio Preto | -18.178 | -43.328 |
| 17 | São Gonçalo do Rio Preto | Rio Preto | -18.199 | -43.340 |
| 18 | São Gonçalo do Rio Preto | Rio Preto | -18.218 | -43.333 |
| 19 | São Gonçalo do Rio Preto | Rio Preto | -18.225 | -43.330 |
| 20 | Diamantina | imprecise locality | -18.238 | -43.611 |
| 21 | Serro | imprecise locality | -18.660 | -43.611 |
| 22 | Alvorada de Minas | Serra do Sapo | -18.720 | -43.611 |
| 23 | Congonhas do Norte | Serra Talhada 1 | -18.760 | -43.771 |
| 24 | Santana de Pirapama | Fechados | -18.786 | -43.878 |
| 25 | Congonhas do Norte | Serra Talhada 2 | -18.811 | -43.755 |
| 26 | Congonhas do Norte | Serra Talhada 2 | -18.880 | -43.760 |
| 27 | Conceição do Mato Dentro | imprecise locality | -19.042 | -43.418 |
| 28 | Santana do Riacho | Lapinha da Serra | -19.110 | -43.670 |
| 29 | Santana do Riacho | Serra do Cipó | -19.210 | -43.489 |
| 30 | Santana do Riacho | Serra do Cipó | -19.257 | -43.544 |
| 31 | Santana do Riacho | Serra do Cipó | -19.266 | -43.182 |
| 32 | Santana do Riacho | Serra do Cipó | -19.267 | -43.547 |
| 33 | Santana do Riacho | Serra do Cipó | -19.268 | -43.581 |
| 34 | Barão de Cocais | Serra da Água Limpa | -19.887 | -43.516 |
| *Bokermannohyla oxente* (outgroup) | | |  |  |
| 0 | Serra do Sincorá | Mucugê, Bahia | -13.005 | -43.705 |
