# Supplementary material for: Past vicariance promoting deep genetic divergence in an endemic frog species of the Espinhaço Range in Brazil: The historical biogeography of Bokermannohyla saxicola (Hylidae)
Source: PLoS One. 2018 Nov 5;13(11):e0206732. doi: 10.1371/journal.pone.0206732 (PMC6218059; doi:10.1371/journal.pone.0206732)
Supplement: S2 Table — (DOC) [file pone.0206732.s002.doc]

**S2 Table.** **Genbank accession numbers of *B. saxicola* samples used in this study.**

|  |  | **Genbank Numbers** | |
| --- | --- | --- | --- |
| **Code Loc** | **Sample** | **COI** | **Cyt-b** |
| 0 | T1335 | MF918839 | MF918625 |
| 7 | AML974 | MF918840 | MF918626 |
| 7 | LMP103 | MF918841 | MF918627 |
| 9 | T420 | MF918842 | MF918628 |
| 6 | TLP273a | MF918843 | MF918629 |
| 6 | TLP273b | MF918844 | MF918630 |
| 6 | TLP832 | MF918845 | MF918631 |
| 5 | HTA303 | MF918846 | MF918632 |
| 5 | HTA304 | MF918847 | MF918633 |
| 5 | HTA305 | MF918848 | MF918634 |
| 5 | HTA307 | MF918849 | MF918635 |
| 5 | HTA308 | MF918850 | MF918636 |
| 5 | HTA309 | MF918851 | MF918637 |
| 5 | HTA314 | MF918852 | MF918638 |
| 5 | HTA318 | MF918853 | MF918639 |
| 5 | HTA319 | MF918854 | MF918640 |
| 5 | HTA320 | MF918855 | MF918641 |
| 5 | HTA321 | MF918856 | MF918642 |
| 5 | HTA323 | MF918857 | MF918643 |
| 5 | HTA333 | MF918858 | MF918644 |
| 5 | HTA334 | MF918859 | MF918645 |
| 5 | HTA336 | MF918860 | MF918646 |
| 5 | HTA340 | MF918861 | MF918647 |
| 5 | HTA343 | MF918862 | MF918648 |
| 5 | HTA363 | MF918863 | MF918649 |
| 5 | HTA367 | MF918864 | MF918650 |
| 5 | HTA393 | MF918865 | MF918651 |
| 1 | BS123 | MF918866 | MF918652 |
| 1 | BS125 | MF918867 | MF918653 |
| 1 | BS127 | MF918868 | MF918654 |
| 1 | BS130 | MF918869 | MF918655 |
| 1 | BS132 | MF918870 | MF918656 |
| 1 | BS133 | MF918871 | MF918657 |
| 1 | BS142 | MF918872 | MF918658 |
| 2 | T179 | MF918873 | MF918659 |
| 2 | T212 | MF918874 | MF918660 |
| 2 | T213 | MF918875 | MF918661 |
| 2 | T214 | MF918876 | MF918662 |
| 2 | T215 | MF918877 | MF918663 |
| 2 | T216 | MF918878 | MF918664 |
| 3 | T421 | MF918879 | MF918665 |
| 3 | T423 | MF918880 | MF918666 |
| 3 | T428 | MF918881 | MF918667 |
| 11 | AML976 | MF918882 | MF918668 |
| 31 | BS001 | MF918883 | MF918669 |
| 31 | BS002 | MF918884 | MF918670 |
| 31 | BS003 | MF918885 | MF918671 |
| 31 | BS004 | MF918886 | MF918672 |
| 31 | BS005 | MF918887 | MF918673 |
| 33 | BS006 | MF918888 | MF918674 |
| 30 | BS007 | MF918889 | MF918675 |
| 30 | BS008 | MF918890 | MF918676 |
| 30 | BS009 | MF918891 | MF918677 |
| 16 | BS010 | MF918892 | MF918678 |
| 16 | BS011 | MF918893 | MF918679 |
| 16 | BS012 | MF918894 | MF918680 |
| 23 | BS013 | MF918895 | MF918681 |
| 23 | BS014 | MF918896 | MF918682 |
| 21 | BS015 | MF918897 | MF918683 |
| 23 | BS016 | MF918898 | MF918684 |
| 23 | BS019 | MF918899 | MF918685 |
| 23 | BS020 | MF918900 | MF918686 |
| 23 | BS021 | MF918901 | MF918687 |
| 23 | BS022 | MF918902 | MF918688 |
| 23 | BS023 | MF918903 | MF918689 |
| 23 | BS024 | MF918904 | MF918690 |
| 32 | BS026 | MF918905 | MF918691 |
| 32 | BS027 | MF918906 | MF918692 |
| 25 | BS031 | MF918907 | MF918693 |
| 25 | BS035 | MF918908 | MF918694 |
| 25 | BS039 | MF918909 | MF918695 |
| 25 | BS041 | MF918910 | MF918696 |
| 25 | BS044 | MF918911 | MF918697 |
| 25 | BS059 | MF918912 | MF918698 |
| 25 | BS062 | MF918913 | MF918699 |
| 25 | BS064 | MF918914 | MF918700 |
| 25 | BS066 | MF918915 | MF918701 |
| 25 | BS068 | MF918916 | MF918702 |
| 18 | BS070 | MF918917 | MF918703 |
| 18 | BS071 | MF918918 | MF918704 |
| 19 | BS073 | MF918919 | MF918705 |
| 19 | BS074 | MF918920 | MF918706 |
| 15 | BS075 | MF918921 | MF918707 |
| 15 | BS077 | MF918922 | MF918708 |
| 15 | BS078 | MF918923 | MF918709 |
| 15 | BS079 | MF918924 | MF918710 |
| 15 | BS080 | MF918925 | MF918711 |
| 15 | BS081 | MF918926 | MF918712 |
| 13 | BS083 | MF918927 | MF918713 |
| 13 | BS086 | MF918928 | MF918714 |
| 18 | BS087 | MF918929 | MF918715 |
| 17 | BS088 | MF918930 | MF918716 |
| 17 | BS091 | MF918931 | MF918717 |
| 15 | BS096 | MF918932 | MF918718 |
| 23 | BS101 | MF918933 | MF918719 |
| 32 | BS104 | MF918934 | MF918720 |
| 15 | BS105 | MF918935 | MF918721 |
| 13 | BS106 | MF918936 | MF918722 |
| 13 | BS107 | MF918937 | MF918723 |
| 15 | BS108 | MF918938 | MF918724 |
| 19 | BS109 | MF918939 | MF918725 |
| 13 | BS110 | MF918940 | MF918726 |
| 19 | BS111 | MF918941 | MF918727 |
| 13 | BS112 | MF918942 | MF918728 |
| 15 | BS113 | MF918943 | MF918729 |
| 13 | BS114 | MF918944 | MF918730 |
| 14 | BS116 | MF918945 | MF918731 |
| 14 | BS117 | MF918946 | MF918732 |
| 14 | BS118 | MF918947 | MF918733 |
| 13 | BS119 | MF918948 | MF918734 |
| 8 | BS158 | MF918949 | MF918735 |
| 8 | BS159 | MF918950 | MF918736 |
| 8 | BS161 | MF918951 | MF918737 |
| 8 | BS162 | MF918952 | MF918738 |
| 8 | BS163 | MF918953 | MF918739 |
| 8 | BS164 | MF918954 | MF918740 |
| 8 | BS165 | MF918955 | MF918741 |
| 10 | BS166 | MF918956 | MF918742 |
| 10 | BS168 | MF918957 | MF918743 |
| 10 | BS171 | MF918958 | MF918744 |
| 10 | BS172 | MF918959 | MF918745 |
| 10 | BS173 | MF918960 | MF918746 |
| 10 | BS174 | MF918961 | MF918747 |
| 10 | BS175 | MF918962 | MF918748 |
| 10 | BS176 | MF918963 | MF918749 |
| 10 | BS177 | MF918964 | MF918750 |
| 10 | BS178 | MF918965 | MF918751 |
| 10 | BS180 | MF918966 | MF918752 |
| 10 | BS181 | MF918967 | MF918753 |
| 10 | BS182 | MF918968 | MF918754 |
| 10 | BS183 | MF918969 | MF918755 |
| 10 | BS184 | MF918970 | MF918756 |
| 10 | BS185 | MF918971 | MF918757 |
| 28 | FL496 | MF918972 | MF918758 |
| 28 | FL497 | MF918973 | MF918759 |
| 28 | FL498 | MF918974 | MF918760 |
| 4 | FL527 | MF918975 | MF918761 |
| 4 | FL601 | MF918976 | MF918762 |
| 4 | FL604 | MF918977 | MF918763 |
| 4 | FL612 | MF918978 | MF918764 |
| 4 | FL620 | MF918979 | MF918765 |
| 4 | FL636 | MF918980 | MF918766 |
| 4 | FL639 | MF918981 | MF918767 |
| 4 | FL672 | MF918982 | MF918768 |
| 4 | FL684 | MF918983 | MF918769 |
| 4 | FL688 | MF918984 | MF918770 |
| 4 | FL690 | MF918985 | MF918771 |
| 4 | FL691 | MF918986 | MF918772 |
| 4 | FL692 | MF918987 | MF918773 |
| 4 | FL693 | MF918988 | MF918774 |
| 4 | FL694 | MF918989 | MF918775 |
| 4 | FL695 | MF918990 | MF918776 |
| 4 | FL703 | MF918991 | MF918777 |
| 4 | FL704 | MF918992 | MF918778 |
| 4 | FL708 | MF918993 | MF918779 |
| 12 | FL759 | MF918994 | MF918780 |
| 12 | FL780 | MF918995 | MF918781 |
| 12 | FL794 | MF918996 | MF918782 |
| 12 | FL797 | MF918997 | MF918783 |
| 12 | FL811 | MF918998 | MF918784 |
| 12 | FL820 | MF918999 | MF918785 |
| 12 | FL875 | MF919000 | MF918786 |
| 12 | FL877 | MF919001 | MF918787 |
| 12 | FL879 | MF919002 | MF918788 |
| 12 | FL883 | MF919003 | MF918789 |
| 12 | FL886 | MF919004 | MF918790 |
| 12 | FL893 | MF919005 | MF918791 |
| 12 | FL895 | MF919006 | MF918792 |
| 12 | FL899 | MF919007 | MF918793 |
| 12 | FL902 | MF919008 | MF918794 |
| 12 | FL906 | MF919009 | MF918795 |
| 12 | FL908 | MF919010 | MF918796 |
| 12 | FL910 | MF919011 | MF918797 |
| 12 | FL912 | MF919012 | MF918798 |
| 12 | FL915 | MF919013 | MF918799 |
| 28 | FLG20 | MF919014 | MF918800 |
| 28 | FLG21 | MF919015 | MF918801 |
| 28 | FLG22 | MF919016 | MF918802 |
| 28 | FLG23 | MF919017 | MF918803 |
| 28 | FLG24 | MF919018 | MF918804 |
| 28 | FLG25 | MF919019 | MF918805 |
| 28 | FLG26 | MF919020 | MF918806 |
| 28 | FLG27 | MF919021 | MF918807 |
| 28 | FLG28 | MF919022 | MF918808 |
| 29 | FLG29 | MF919023 | MF918809 |
| 29 | FLG30 | MF919024 | MF918810 |
| 29 | FLG31 | MF919025 | MF918811 |
| 29 | FLG32 | MF919026 | MF918812 |
| 24 | HTA002 | MF919027 | MF918813 |
| 24 | HTA003 | MF919028 | MF918814 |
| 24 | HTA004 | MF919029 | MF918815 |
| 24 | HTA005 | MF919030 | MF918816 |
| 11 | LMP102 | MF919031 | MF918817 |
| 28 | PPGT136 | MF919032 | MF918818 |
| 27 | T104 | MF919033 | MF918819 |
| 27 | T105 | MF919034 | MF918820 |
| 20 | T419 | MF919035 | MF918821 |
| 26 | T70 | MF919036 | MF918822 |
| 26 | T71 | MF919037 | MF918823 |
| 26 | T72 | MF919038 | MF918824 |
| 26 | T73 | MF919039 | MF918825 |
| 26 | T75 | MF919040 | MF918826 |
| 26 | T77 | MF919041 | MF918827 |
| 34 | TLP792 | MF919042 | MF918828 |
| 34 | TLP793 | MF919043 | MF918829 |
| 34 | TLP794 | MF919044 | MF918830 |
| 34 | TLP795 | MF919045 | MF918831 |
| 34 | TLP796 | MF919046 | MF918832 |
| 34 | TLP797 | MF919047 | MF918833 |
| 34 | TLP798 | MF919048 | MF918834 |
| 34 | TLPG256a | MF919049 | MF918835 |
| 34 | TLPG256b | MF919050 | MF918836 |
| 34 | TLPG256c | MF919051 | MF918837 |
| 22 | UFMGG547b | MF919052 | MF918838 |
