# Supplementary figures and images for: Past vicariance promoting deep genetic divergence in an endemic frog species of the Espinhaço Range in Brazil: The historical biogeography of Bokermannohyla saxicola (Hylidae)
Source: PLoS One. 2018 Nov 5;13(11):e0206732. doi: 10.1371/journal.pone.0206732 (PMC6218059; doi:10.1371/journal.pone.0206732)

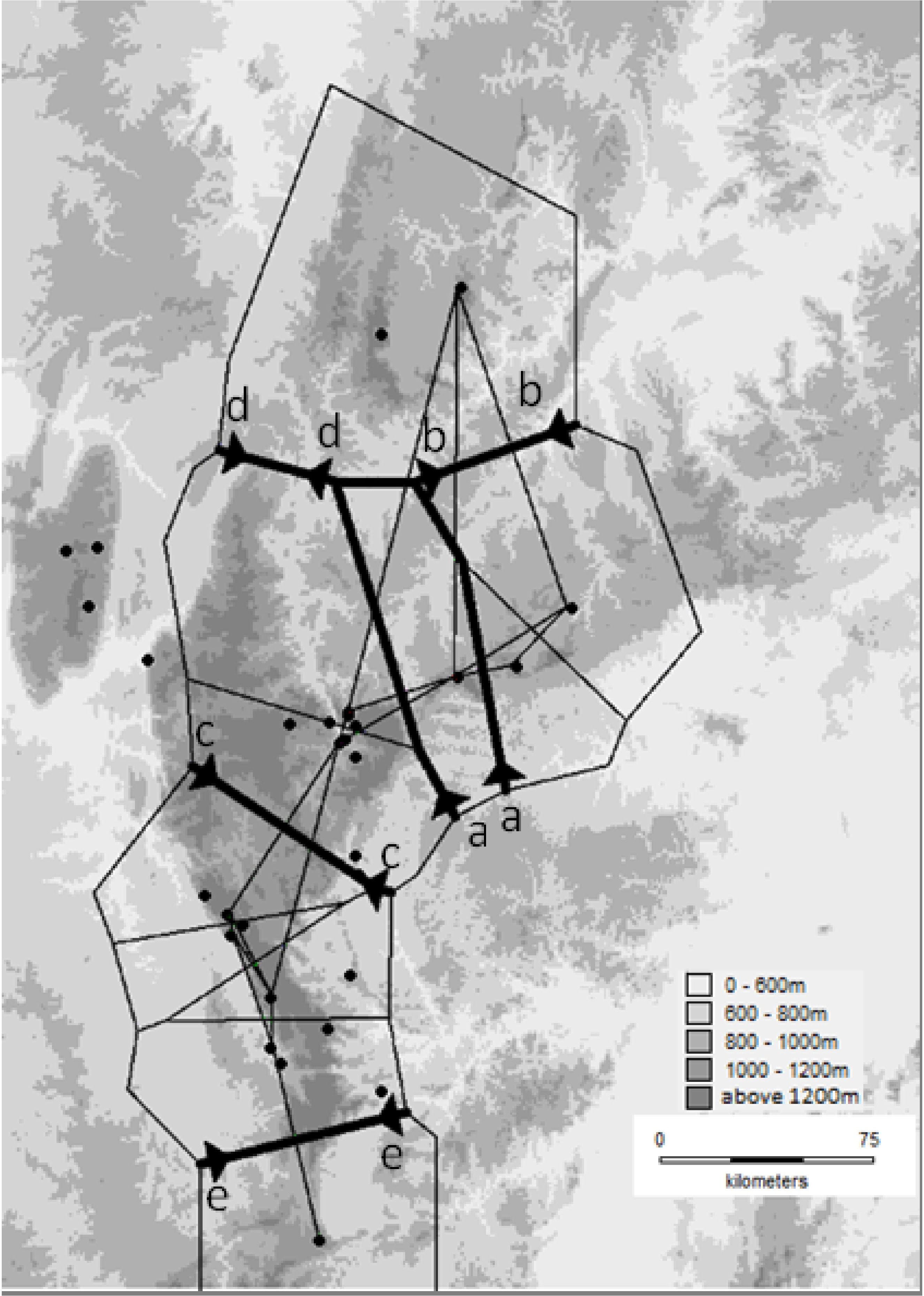

Supplement: S1 Fig — Voronoi tessellation and Dealuney triangulation superimposed to the sample map. The five barriers are identified by alphabetical order at the edges of each barrier. (TIF) [file pone.0206732.s003.tif]
